# Design of Experiments for Dynamic Test Runs in Solvent-Based CO_2_ Capture Pilot Plants

**DOI:** 10.1021/acs.iecr.5c01802

**Published:** 2025-09-29

**Authors:** Anderson Soares Chinen, Joshua C. Morgan, Debangsu Bhattacharyya, Benjamin Omell, Michael Matuszewski

**Affiliations:** a Department of Chemical and Biomedical Engineering, 5631 West Virginia University, Morgantown, West Virginia 26506, United States; b 17213 National Energy Technology Laboratory, 3610 Collins Ferry Road, Morgantown, West Virginia 26507, United States; c 17213 National Energy Technology Laboratory, 626 Cochrans Mill Road, Pittsburgh, Pennsylvania 15236, United States

## Abstract

Test runs in the pilot plants consume significant resources, and therefore, the learning from test runs should be maximized. Test runs conducted in the pilot plants are often steady state. It takes several hours for reaching steady-state in the pilot plants, and thus, the duration of the test runs needs to be long even for collecting few steady-state data points. On the other hand, a large number of measurements can be collected through dynamic test runs in a short span of time. This paper presents a systematic design of dynamic experiments (DoDEs) for identifiability of model parameters, which is achieved by persistently exciting the inputs signals. A pseudorandom binary sequence (PRBS) is designed as the input signal for DoDE due to its efficiency in obtaining sufficient spectral content. However, due to the long sequence size of the PRBS signal, a Schroeder-phase input signal, which is a multisine signal, is also designed. Tests for both types of signals are run in the Pilot Solvent Test Unit (PSTU) at the National Carbon Capture Center in Wilsonville, Alabama. The transient data are used to solve dynamic data reconciliation and parameter estimation problem. The estimated parameters are found to be not only superior to those estimated from using data collected from hundreds of steady-state test runs in a nonreactive (air–water) system, but the parameters could be estimated by using the dynamic data collected for about 24 h from the pilot plant for the MEA-H_2_O–CO_2_ system.

## Graphical abstract

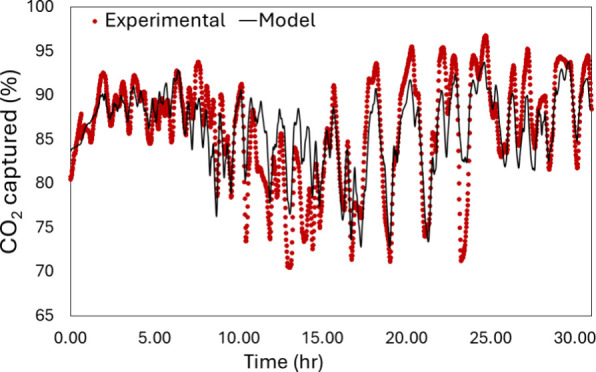

## 1 Introduction

Since 1751, over 400 billion metric tonnes of carbon has been released to the atmosphere from the combustion of fossil fuels and cement production alone.^1,2^ As it is anticipated that the human civilization will continue to use a significant amount of fossil fuels in the foreseeable future, postcombustion CO_2_ capture and sequestration (CCS) technologies are expected to play a leading role in reducing greenhouse gas emissions.^3,4^

Most of the test runs in the pilot plants for solvent-based capture units are steady-state.^5–11^ Dynamic test runs for solvent-based CO_2_ capture processes, especially in the pilot plants, are not common. Kvamsdal et al. (2011) performed a series of open-loop step tests in the inlet gas CO_2_ concentration at the Validation of Carbon Capture (VOCC) pilot-plant for model validation.^12^ Faber et al. (2011) conducted tests in the Esbjerg pilot plant for studying open loop responses.^13^ The authors concluded that the plant takes about 1 h and 30 min, on an average, to reach the new steady-state after a step change in either flue gas flow rate, lean MEA flow rate, or reboiler duty is introduced. Flo̷ et al. implemented a series of step-changes in solvent or steam flow rates at the Brindisi pilot-plant,^14^ and at the Glo̷shaugen pilot-plant^15^ to generate data for model validation and better understanding of the transients.

Montañés et al. performed dynamic step tests in the flue gas flow rate at Technology Center Mongstad (TCM) for model development and validation.^16^ The validated model was then used to perform a subsequent study for evaluating the performance of decentralized control structures for flexible operations.^17^ Other dynamic test runs in pilot plants for CO_2_ capture have been conducted for varieties of objectives- at the Naasvlakte power station for validating an equilibrium based dynamic model;^18^ at the CSIRO pilot-plant for verifying the viability of flexible operations and providing data for model validation;^19^ at the Sulzer Chemtech’s facilities for studying flexible operation;^20^ at the Pilot-scale Advanced Capture Technology (PACT) facilities of the United Kingdom Carbon Capture and Storage Research Centre (UKCCSRC), for studying various start-up procedures.^21^

In all the test runs noted above, either only a single step change for only one variable at a time has been implemented or few step tests have been conducted without considering any design objective for the input signals. One of the issues of a single step change is that it is persistently exciting only for only a first order system.^22^ Since the CO_2_ capture system is of higher-order, a single step change cannot ensure the persistence of excitation. The authors of this paper have previously performed test runs at the National Carbon Capture Center (NCCC) to address this issue.^23^ In that work, it was intended to implement pseudorandom binary sequence (PRBS) signals for maintaining persistence of excitation. However, since PRBS test runs needed to be programmed in the plant distributed control system and only limited time was available for designing and implementing the dynamic test runs, only a quasi-PRBS signal could be implemented.^23^ The quasi-PRBS signal lacked the properties of the full-PRBS signal. That study investigated transient response by considering three input variables: flow rates of solvent, flue gas, and reboiler steam. Pseudo-PRBS test runs were implemented for one variable at a time since the worst-case dynamic response that could occur as a result of simultaneous variation in all three variables could not be estimated, and thus acceptability to the plant equipment items and operating personnel could not be established. Therefore, the data did not capture the confounding effect of all three variables. Furthermore, duration of each of those test runs were constrained so that the data captures the effect of the single input variable being investigated on either the absorber or stripper. That was done to minimize the effects of the changing boundary conditions such as the concentration and loading of the lean solvent. However, in real life these effects cannot be avoided, and therefore it is desired that the dynamic model can represent such interactions.

In summary, the literature review shows the following limitations in the existing literature on dynamic test runs:

- A test run designed with due consideration of the order of the CO_2_ capture system has not been implemented yet. In the design of dynamic experiments (DoDE) developed in this work, a systematic approach is developed for designing test signals to ensure persistence of excitation of the underlying CO_2_ capture system.
- Confounding effects of multiple inputs in CO_2_ capture systems are not captured in most of the studies in the literature.^24,10,11^ It should be noted that in a general nonlinear system such as the CO_2_ capture process, impacts of the input variables on the output variables are not additive unlikely of the linear systems and therefore impact of changes of multiple inputs simultaneously should be captured in the transient data. In this work, we have considered four input variables-flow rates of solvent, flue gas, and reboiler steam, and CO_2_ concentration in the incoming flue gas. Input signal designs for all four variables are done perturbing them simultaneously.
- In real-life, implementation error in the designed input signal is unavoidable. Furthermore, the real-life data can be noisy and often do not satisfy mass and energy balances. These aspects are hardly addressed in the literature in this area. A dynamic data reconciliation framework has been developed and implemented in this work.^23^
- Existing pilot plant test runs for CO_2_ capture have been mainly performed for model validation or studying dynamics or flexible operation. In this work, the objective of the DoDE is to ensure identifiability of model parameters by persistently exciting the inputs signals. For estimating the parameters using the plant transient data, a dynamic data reconciliation and parameter estimation framework is developed in this work and used to estimate the parameters corresponding to the holdup model.
- For transient studies where multiple inputs are perturbed simultaneously, the resulting worst-case outputs may not be acceptable as they can cause equipment damage, lead to safety issues, and violation of operating constraints. Therefore, the input signals should be ‘plant-friendly’, i.e., the resulting changes in all variables are acceptable to the plant personnel. While various input signal designs can satisfy the objectives of the dynamic experiments as noted above, their plant-friendliness properties can be different. In this work, two different input signals, namely the PRBS design and a Schroeder-phased signal with zippered power-spectra, are designed with due consideration of their plant-friendliness properties. For designing plant-friendly input signals, the crest factor, or the ratio between the current value of the signal and its maximum value, is evaluated. Failure to satisfy design constraints can result in signal design that is unacceptable due to variability, frequency content (harsh changes), amplitude (designed values cannot be achieved at implementation) and waveform (some signal forms may not be implementable in a given control system).
- Pilot plant experiments can cost considerable resources, money and time and therefore the duration of such test runs should be minimized. It is possible that there are multiple input signal designs that can satisfy the design objectives, but the time taken for executing these signals may be considerably different. In this work, two different designs are developed and their performance as well as the duration are evaluated.

This work was undertaken as part of the United States Department of Energy’s Carbon Capture Simulation Initiative (CCSI) that is focused on development of a suite of computational tools and models (CCSI Computational Toolset) to accelerate the development and commercialization of CO_2_ capture processes.^25^ Its successor, the Carbon Capture Simulation for Industry Impact (CCSI^2^) aims to simultaneously expand the capabilities of the CCSI Computational Toolset while utilizing it to provide R&D support for specific projects related to development and demonstration of novel CO_2_ capture technologies.

Previous test runs undertaken by the U.S. Department of Energy’s CCSI/CCSI^2^ team in the National Carbon Capture Center (NCCC) on the MEA-based CO_2_ capture process were performed for model validation by using a space-filling approach for the design of experiments.^10^ Later, a rigorous sequential DoE was also developed for minimizing the maximum variance in the predicted values. Both of these campaigns were operated under steady-state regimes.^11^ A DoDE has been presented earlier by the authors of this paper, where a data-driven approach was used due to the nonavailability of a process model at that time.^23^ In addition, due to the lack of available time, only a quasi-pseudo random binary signal (PRBS) was implemented. Furthermore, in those test runs, each input variable was investigated independently. Duration of those test runs was also limited, so that either the absorber or stripper dynamics were observed depending on what input variable was perturbed. This paper significantly extends the DoDE by developing a fully systematic method where a model-based approach is used to design the DoE by considering the multivariable system with due evaluation of the plant-friendliness of the design signals. Duration of the test runs also captured the effect of the integrated system. Furthermore, the test run data are processed, reconciled, and used for parameter estimation.

## 2 Pilot Plant Set up and Process Model

A simplified process flow diagram (PFD) of the NCCC pilot plant is shown in Figure 1. The figure also shows the regulatory controllers for the flow, pressure, level, and temperature. During DoDE, lean solvent flow rate, flue gas flow rate, and steam flow rates were used as input variables. Another input variable that was considered is the flue gas CO_2_ concentration, that was varied by manipulating the dilution air flow rate injected to the incoming flue gas from the power plant. It should be noted that while the stripper reboiler outlet temperature or temperature at a specific location in the stripper is typically controlled by manipulating the steam flow rate to the reboiler, during dynamic test runs conducted in this work, the stripper temperature is not controlled; rather, the reboiler steam flow rate itself is considered to be the degrees of freedom (DOF). The reason for selecting these four input variables is the following. Level controllers shown in Figure 1 simply maintain the system inventory, so they are not suitable for being used as DOF. Pressure of the absorber could not be changed due to limitation in the flue gas blower design. The stripper top pressure remained near atmospheric as any increase would lead to a higher stripper bottom temperature that would accelerate solvent degradation. The temperature controllers are for the water coolers, where only negligible variation could be made in their set point given the incoming temperature of the cooling water from the plant cooling tower. Therefore, the setup and set point of all regulatory controllers other than those used as inputs were left at their typical set points. More information about the NCCC pilot plant configuration, its control configuration, operation, and sampling and measurement can be found in our earlier publications.^10,11,23^

**Figure 1:**
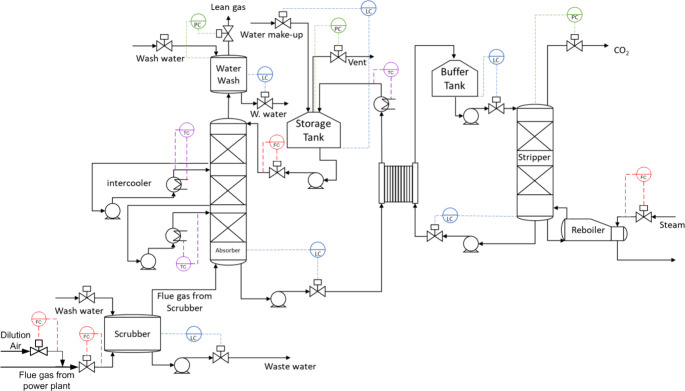
Simplified PFD of the NCCC pilot-plant. Orange-colored controllers represent the flow controllers utilized in the test-protocol. Blue-colored, purple-colored, and green-colored controllers represent the level, temperature, and pressure controllers.

The process model of the MEA-H_2_O–CO_2_ system used in this work has been described in detail in our earlier works.^10,11,23,26–28^ In summary, models were developed and their parameters were optimally estimated for thermo-physical properties such as density, viscosity, and surface tension by using the experimental data.^26^ The vapor–liquid equilibrium (VLE) model as well as enthalpy, and chemistry models were developed and their key parameters were regressed simultaneously by using VLE, heat capacity and heat of absorption data for binary (MEA-H_2_O) and ternary (MEA-H_2_O–CO_2_) systems.^27^ In addition, an integrated approach was developed for development and simultaneous parameter estimation of kinetic submodels, mass transfer coefficient submodels, and interfacial area submodels by using the experimental data from the wetted wall columns and structured packings.^28^ Hydraulic submodels that include models for liquid holdup and pressure drop were also developed and their parameters estimated by using experimental data from structured packings.^28^ Earlier works also include development of the steady-state models for the NCCC pilot plant^10,11^ as well as the dynamic model^19^ that is used in this work for generating the test runs. Parameters of this dynamic model are updated using the dynamic data collected in this work through DoDE. The updated dynamic model is then used to run several case studies. The dynamic model has been developed in Aspen Plus Dynamics (APD) by representing the plant configuration as well as the control setup including the set points of the respective regulatory controllers. Solvent degradation has been ignored in these models, which is expected to be negligible during the dynamic test runs that spanned less than 3 days in total. For more details, interested readers are referred to the works cited in this paragraph as well as our other publications that provide additional details.^29,30^

## 3 Dynamic Design of Experiments

The dynamic data can capture the effect of some models/correlations that may be difficult or confounded in the steady-state data. For example, let us consider the following dynamical system given by eq 1:

H(η,y,u,θ)η̇=f(η,y,u,θ);⁣g(η,y,u,θ)≤0 1

where $\eta \in R^{N_{\eta }},y\in R^{N_{y}},u\in R^{N_{u}},\theta \in R^{N_{\theta }}$ denote the differential variables, algebraic variables, input variables, and parameters, respectively. For CO_2_ capture systems using solvents in an absorber-stripper system, where the differential equations correspond to the materials, momentum, and energy conservations, the corresponding holdup term, denoted by *H*(η, *y*, *u*, θ) in eq 1, directly affects the transient response of the system. Thus, the parameters corresponding to these holdup models can be estimated using the transient data. In general, the holdup may or may not affect the steady-state data or may affect multiple measured variables in a way that it is difficult or cumbersome to estimate the corresponding parameters. In a solvent-based CO_2_ capture system using a packed tower, the holdup affects the reaction rate and the pressure drop.^28^ Therefore, to estimate the parameters corresponding to the holdup model, either additional experimental tests need to be performed for estimating the change in the holdup as a function of physical properties and operating conditions in absence of reactions or ‘known’ reaction rates or it needs to be simultaneously estimated along with the parameters corresponding to the pressure drop and reaction rate model thus causing potential lack of identifiability.

The objective of the DoDE in this work is to ensure identifiability of model parameters, rather than gathering data for the validation of a dynamic model, or for understanding the pilot-plant behavior under rapid load changes as was done in our earlier work.^23^ It should be noted that while steady-state design of experiments can be developed for ensuring identifiability of model parameters, the key issue is the lack of information or convoluted information in the steady-state data about the material and thermal holdup as the time derivative in eq 1 becomes zero at steady-state. For ensuring identifiability of model parameters by using the transient data, input signals should be designed to ensure persistence of excitation.^22^ While signals of different frequency content and waveforms can help to achieve the required properties,^31^ following aspects need to be taken into consideration while selecting and designing the signals:

- Variability: it is correlated to the level of excitation of the signal, and may cause the output being studied to reach an undesired value or specification.
- Frequency content: high frequency in the input signal may damage valves and/or other equipment items.
- Amplitude: high amplitudes of the input signals may be unacceptable and can lead to unacceptable change in the actuators whereas low amplitudes may result in low signal-to-noise ratio for the output variables.
- Waveform: some waveforms may be difficult to program in the plant control system or can have large implementation error.

The power spectrum of a signal is an important criterion for designing the input signals. It shows how the power of a signal is distributed over the frequencies. For a discrete-time signal *x*(*n*) being applied to a process of order *n*, the power spectrum can be calculated from its discrete Fourier transform (DFT) by using eqs 2 and 3:

$$X\left(k\right)=\frac{1}{N}\sum_{n=0}^{N-1}x\left(n\right)W_{N}^{kn}$$ 2

$$W_{N}^{kn}=e^{-j2\pi kn/N}$$ 3

This transformation is often called “forward DFT”, while a “backward DFT” differs by the sign of the exponent in eq 3). The DFT can be readily calculated using the fast Fourier transform (FFT) algorithm. Since the designed input signals should be real, the output signal *X*(*k*) satisfies the symmetry given by eq 4:

$$X_{m}\left(k-n\right)=X^{*}\left(n\right)$$ 4

where * denotes complex conjugate. The power spectrum (PS) can then be generated as follows by using eq 5:

$$\mathrm{PS}\left(f_{m}\right)=2\frac{{\left[X_{m}\right]}^{2}}{f_{S}N},,m=0,\cdot \cdot \cdot ,N/2$$ 5

The power spectrum can be expressed in decibels (dB) to normalize its value and give an overall representation of its intensity (eq 6):

$$\mathrm{ratio}\;\left[\mathrm{dB}\right]=10\;\log_{10}\left(\frac{\mathrm{signal}\;\mathrm{power}}{\mathrm{reference}\;\mathrm{power}}\right)$$ 6

Another important design criterion for the input signal is the sampling frequency. If a signal has its maximum frequency *f*_Ny_, then as per the Nyquist theorem, its sampling frequency *f*_*s*_, should be at least: *f*_*s*_ = 2*f*_Ny_.

It is desired that the parameter estimates be unbiased, i.e., *E*{θ̂_*N*_} = θ_*N*_ for a set of parameters θ̂_*N*_ and consistent, i.e., $\lim_{n\to \infty }{\mathrm{\theta ̂}}_{N}=\theta_{N}$ . It is desired that θ̂_*N*_ has a small covariance. It is also desired that the estimator is efficient, i.e., its covariance is at least equal to the Cramér–Rao lower bound (CR), which is given by eq 7:

$$\mathrm{CR}={Fi}^{-1}\left(\theta_{N}\right)$$ 7

where *Fi* is the Fisher information matrix, which is a measure of the information content in the experimental data.

To satisfy the objectives discussed above, two signals, the PRBS and the Schroeder-phased input waveform signals, are considered for the DoDE implemented at the NCCC pilot plant.

### 3.1 Overall Design Methodology

A summary of the input signal design approach is described by the algorithm presented in Figure 2. For each input signal design technique, various decisions need to be made and information needs to be obtained as listed below by using a plant model or by performing preliminary step tests in the plant:

- Number of input variables
- Feasible ranges of input variables
- Rate of change of input variables
- Maximum settling time
- Lowest dominant time constant
- Highest dominant time constant
- Factors and variables for plant-friendliness analysis

**Figure 2:**
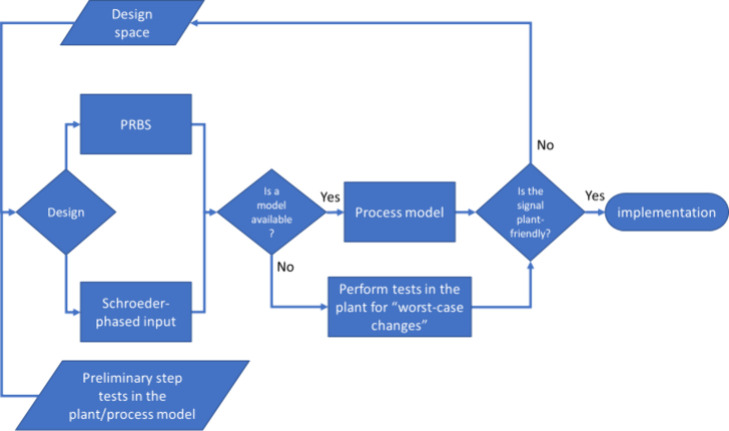
DoDE Development Approach.

For plant-friendliness analysis, plant operating personnel need to be consulted and/or hardware limitations and operating constraints need to be checked with due consideration of the safety, environmental emission, unmodeled phenomena, etc. While input plant-friendliness can be ensured through a proper design, it is hard to ensure output plant-friendliness without a reasonably good preliminary model. In the absence of a model, a conservative design may need to be developed.

### 3.2 Pseudorandom Binary Signal Design

A PRBS is a two-level signal, represented by 0’s or 1’s generated with shift registers, which is given by eq 8:^32^

$$u\left(t\right)=\mathrm{rem}\left(A\left(q\right)u\left(t\right),2\right)=\mathrm{rem}\left(a_{1}u\left(t-1\right)+\cdot \cdot \cdot +a_{n}u\left(t-n\right),2\right)$$ 8

where rem­(*x*, 2) is the remainder of *x* divided by 2, which provides a binary value.

The PRBS is a deterministic signal that has covariance similar to the white noise, but its power spectrum is an impulse train, while the noise has a flat spectrum. The signal has a length *N*_s_, with the design equations given by eqs 9–14:

$$N_{s}=2^{n_{r}}-1$$ 9

$$N_{\mathrm{s1}}\geq \frac{6\pi \tau_{\mathrm{dom}}^{H}}{T_{\mathrm{sw}}}$$ 10

$$N_{\mathrm{s2}}\geq p\times D$$ 11

$$N_{s}=\max\left(N_{\mathrm{s1}},N_{\mathrm{s2}}\right)$$ 12

$$D=\frac{T_{\mathrm{settle}}^{\max}}{T_{\mathrm{sw}}}$$ 13

$$T_{\mathrm{sw}}\leq \frac{2.8\tau_{\mathrm{dom}}^{L}}{2}$$ 14

In eqs 9–14, *n*_r_, τ_dom_^H^, τ_dom_^L^, *T*_sw_, and *T*_settle_^max^ denote the number of shift registers, fastest and slowest time constants, switching time, and maximum settling time, respectively. It can be noted that for designing the PRBS, the time constants can be estimated by using a process model, if available or through step tests in an actual plant. For example, during the 2014 NCCC test runs conducted by the CCSI team, the slowest (τ_dom_^H^) and fastest (τ_dom_^L^) time constants for the NCCC pilot plant were estimated through preliminary step tests.^23^ Similarly, *T*_settle_^max^ is estimated by considering settling time of the output variables and obtaining the maximum out of them. This can be obtained in the similar manner like time constants by using a preliminary model or by performing step tests in the plant. For multivariable PRBS design, it should be ensured that there is lack of cross-correlation between the signals.^33^ This is achieved by introducing a delay *D* between the subsequent design variables until all *p* variables are exhausted. The delay can be calculated by taking into account the switching time *T*_sw_ as shown in eq 13). The overall time required to implement the signal for a single variable can be obtained by multiplying the signal length *N*_*s*_ with the switching time, *t*_final_ = *T*_sw_ *N*_s_. A persistently exciting input signal with high signal-to-noise ratio may not be acceptable to the plant due to its possible impact on equipment wear and tear, product quality violation, safety hazards, and violation of environmental hazards. Therefore, the signals need to be designed such that the sequence is ‘plant-friendly’.^34,35^ The friendliness of the input signal can be quantified by considering the crest factor, which can be represented by the ratio of the *L*_∞_ norm to the *L*_2_ norm of the signal. In addition to the plant-friendliness of the input signals, it was also desired that the output plant-friendliness be considered by taking into account the variability in the CO_2_ capture percentage. It can be noted that a large variability in the CO_2_ capture rate can cause a loss in the solvent from the absorber due to large variations in the pressure. The magnitudes of the bilevel input signals are adjusted to obtain the desired boundedness in the variability of the output. All other parameters as discussed above for PRBS design were determined by simulating the APD model described in our earlier work.^23^ Due to expected inaccuracies in the model, the designed signals were implemented in the NCCC control system in such a way that the signal magnitude for each bilevel signal could be readily changed if violation of the plant friendliness criteria was observed.

The signal magnitudes of each PRBS input were constrained as follows based on the NCCC design:

- Lean solvent flow rate: 5400–5950 kg/h
- Flue gas flow rate: 2130–2360 kg/h
- Flue gas CO_2_ concentration: 10–17.5 wt %
- Steam flow rate: 465–515 kg/h

Figure 3 presents all the signals as implemented in NCCC. For ease of representation, individual signals are scaled so that the relative time delays can be clearly seen in Figure 3. The gas flow rates, solvent flow rates, steam flow rates, and flue gas CO_2_ concentration are scaled between −1 to 1, 2 to 4, 5 to 7, 8 to 10, respectively. In terms of real values, these values correspond to 2150, 2350, 5400, 5900, 470, and 510 kg/h, respectively.

**Figure 3:**
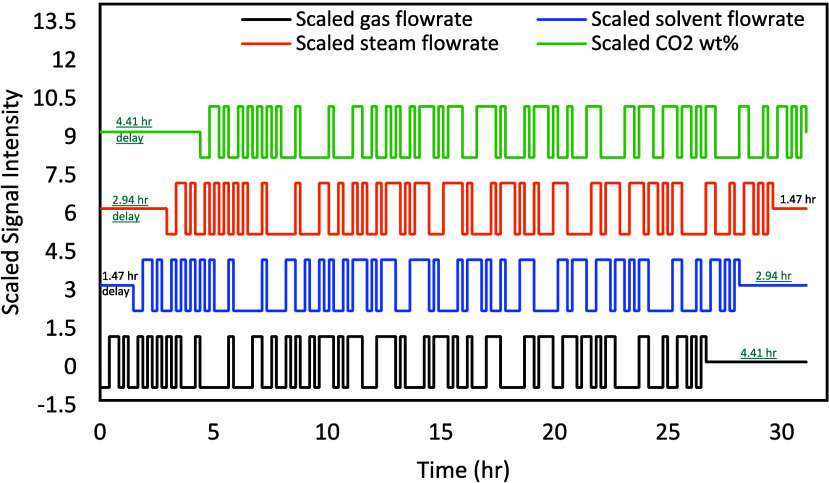
Scaled PRBS design for implementation in the pilot plant (Signals are scaled so that the signals and the time delay can be distinct; scaled values do not intend to show the relative intensity of the signals).

As all of the inputs were changed simultaneously as shown in Figure 3, it was desired to evaluate the worst-case variability in the output. The output plant-friendliness analysis was done by using the APD model presented in our previous work.^23^ The results are shown in Figure 4. Based on the switching time, it was determined that if the maximum change in the CO_2_ capture is constrained between 65 and 90%, it would result in an acceptable rate of change in the pilot plant. During implementation in NCCC, no changes in the input signals were necessary, and the designed signals as shown in Figure 3 could be implemented as is despite the implementation error as will be described later.

**Figure 4:**
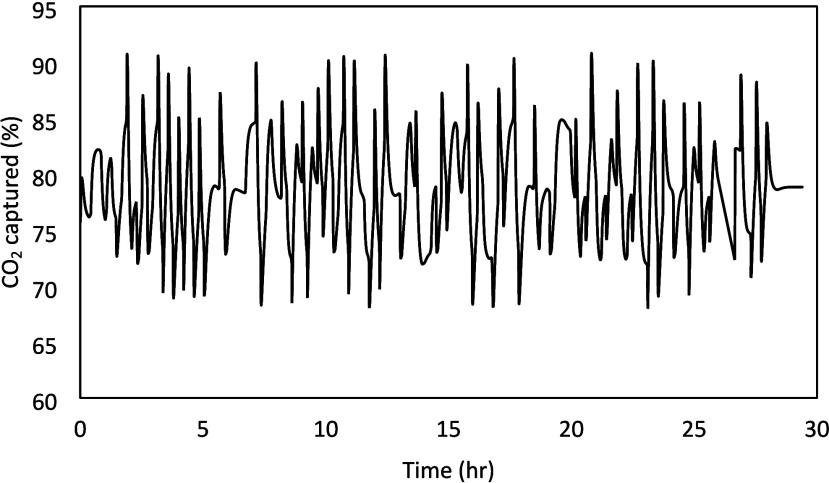
Estimated CO_2_ capture prediction from the PRBS designed signals.

Figure 5 presents the power spectrum of the designed PRBS signal, and Figure 6 presents the power spectrum of the CO_2_ capture output signal. It can be observed that the power of the signals is reasonably uniformly distributed over the entire frequency range of interest and sufficiently positive, which are desired design criteria.

**Figure 5:**
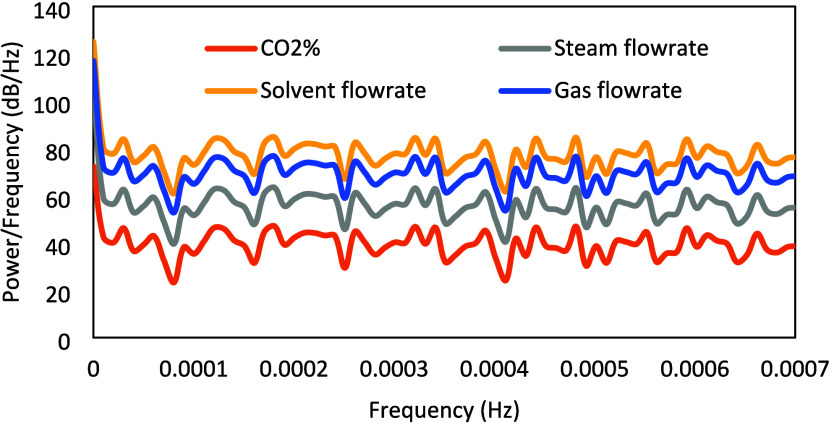
Power spectrum of PRBS DoDE.

**Figure 6:**
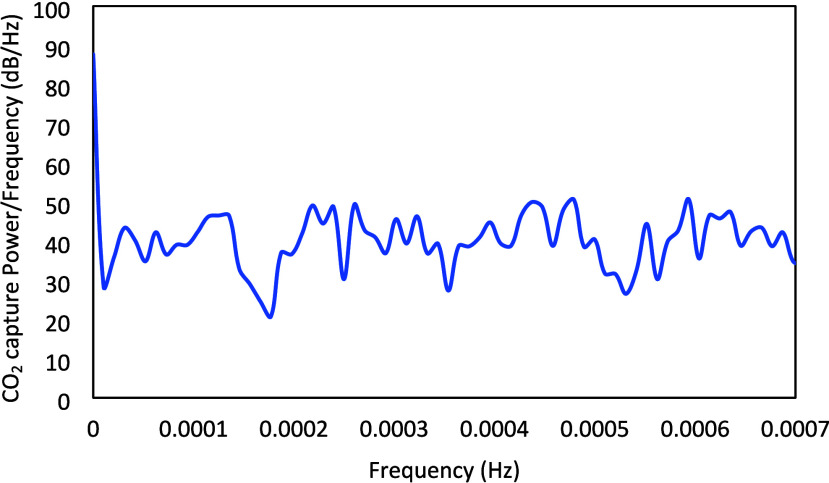
Power spectrum of simulated CO_2_ capture corresponding to the designed PRBS signals.

### 3.3 Schroeder-Phased Input Signal Design

The Schroeder-phase input is a periodic and deterministic multisine input (eq 15):

$$\begin{array}{l} u_{n}=\lambda_{n}\sum_{j=1}^{N_{s}/2}\sqrt{2\alpha_{\left[n,j\right]}}\;\cos\left(\omega_{j}kT+\Phi_{\left[n.j\right]}\right),\\ k=1,2\cdot \cdot \cdot ,N_{s} \end{array}$$ 15

It has a flexible spectrum that can be designed based on the specific requirement of a given process, as the input energy can be placed at discrete frequencies and therefore, low peak-factors, or crest factors can be attained. Equations 16–21 are used for designing it:

$$\omega_{j}=\frac{2\pi j}{N_{s}T}$$ 16

$$\alpha_{\left[n,j\right]}=\{\begin{array}{ll} 1, & j=L_{u}\left(i-1\right)+n\\ 0, & \mathrm{otherwise} \end{array}$$ 17

$$\frac{2\pi }{N_{s}T}\leq \omega \leq \frac{2\pi n_{s}L_{u}}{N_{s}T}<\frac{\pi }{T}$$ 18

$$\max\left(2n_{s}L_{u},\frac{2\pi }{\omega_{*}T}\right)\leq N_{s}\leq \frac{2\pi n_{s}L_{u}}{\omega^{*}T}$$ 19

$$\frac{1}{3\tau_{\mathrm{dom}}^{H}}=\omega_{*}\leq \omega \leq \omega^{*}=\frac{2}{\tau_{\mathrm{dom}}^{L}}$$ 20

$$n_{s}\geq \frac{1}{L_{u}}\frac{\omega^{*}}{\omega }$$ 21

In eqs 15–21), λ_*n*_ is a scaling factor, *N*_s_ is the signal period, *T* is the sampling time, ω_*j*_ denotes the frequency, and Φ_[*n*.*j*]_ denotes the phase of sinusoids. The coefficient α_[*n*,*j*]_ is used to specify the power of each sinusoid. The coefficients α_[*n*,*j*]_ are determined by assuming that the signals are orthogonally excited in frequency. In this work, a “zippered” design is used for the coefficients,^36^ which means that when a specific Fourier coefficient is nonzero along the frequency range, all other Fourier coefficients are zero-valued making the individual signals independent and uncorrelated. The total number of harmonics is determined by the number of excited sinusoids *n*_s_ and the number of input variables *L*_u_ using the equation *n*_h_ = *n*_s_ *L*_u_.

The phase matrix Φ_[*n*.*j*]_ provides considerable flexibility to design the Schroeder-phased signal. A simple general form for specifying the phases requires consideration of the relative power *p*_*j*_ of the *j*th harmonic as given by eqs 22 and 23:^34^

$$\sum_{j=1}^{N}p_{j}=1$$ 22

$$\Phi_{j}=2\pi \sum_{k=1}^{j}p_{k}$$ 23

The design parameters, such as the signal period *N*_s_ and the lower and upper frequency bounds ω_*_ and ω*, are calculated by simulating the APD model of the NCCC pilot plant.^23^ The corresponding signals for the four inputs are presented in Figure 7. It can be noted that this plot is generated using λ = 1, for demonstrating the issue with the high crest factor if the signals are properly designed. The final values of λ_*n*_ are determined to ensure the signal would be constrained under the same operating conditions as described earlier for the PRBS signal design. The high peaks, i.e., high crest factors, observed at *t* = {6, 12, 18} h are not acceptable. To make the signals plant-friendly, the phases Φ_[*n*.*j*]_ can be designed appropriately.

**Figure 7:**
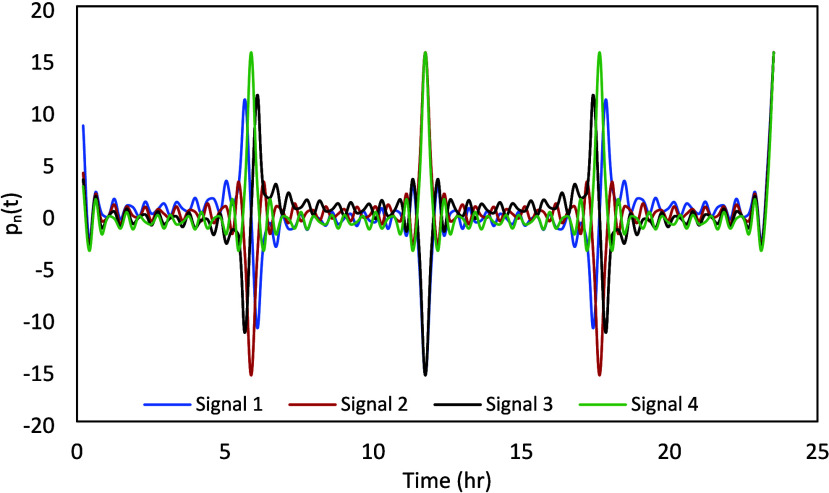
Nonoptimized Schroeder-phased design signals.

To avoid the issue with high crest factor (CF), Φ_[*n*.*j*]_ was determined by minimizing CF, as defined in eqs 24–26:^37^

$$\mathrm{CF}=\frac{L_{\infty }\left(p_{n}\right)}{L_{2}\left(p_{n}\right)}$$ 24

$$L_{\infty }\left(p_{n}\right)=\max\left|p_{n}\right|$$ 25

$$L_{2}\left(p_{n}\right)={\left[\frac{1}{T}\int_{0}^{T}{\left|p_{n}\left(T\right)\right|}^{2}\;dT\right]}^{1/2}$$ 26

The signals obtained for each of these variables are presented in Figure 8. While designing the signal for the CO_2_ concentration in the flue gas, its variability is kept constrained based on the existing control strategy in NCCC, where the flue gas CO_2_ concentration is controlled by diluting it with air. It should be noted that these signals were simultaneously implemented, as shown in Figure 9.

**Figure 8:**
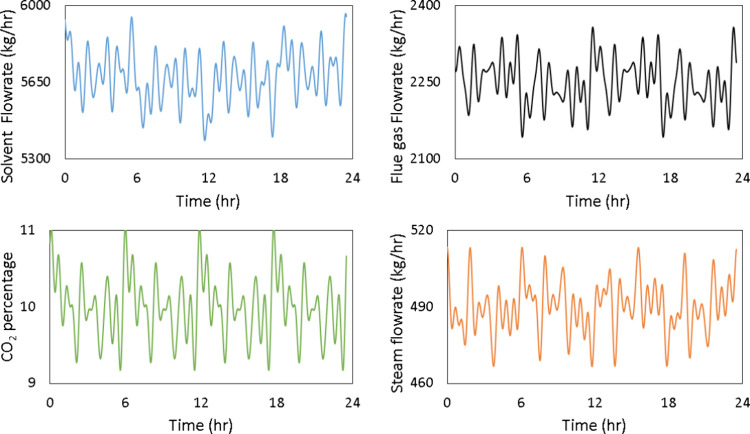
Designed Schroeder-phased input for solvent flow rate, flue gas flow rate, CO_2_ concentration in the flue gas, and steam flow rate.

**Figure 9:**
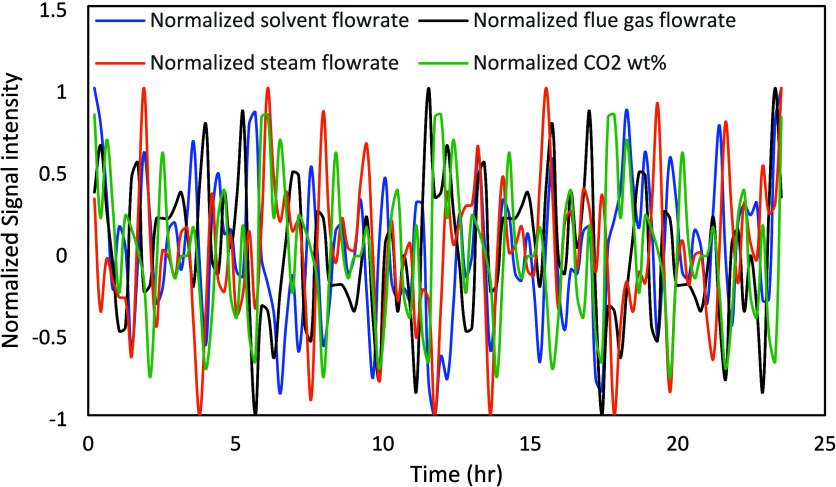
Combined normalized signals for the Schroeder-phased design.

Figure 10 presents the estimated CO_2_ capture percentage from the Schroeder-phase input signal calculated using the existing Aspen Plus Dynamic model. It can be observed that the CO_2_ capture would be expected to remain constrained within the bounds of 65–90% as desired. In addition, the worst case changes and rate of change were found to be acceptable. Similar to the PRBS signal, these signals were implemented in the NCCC control system in such a way that it could be modified readily, if needed.

**Figure 10:**
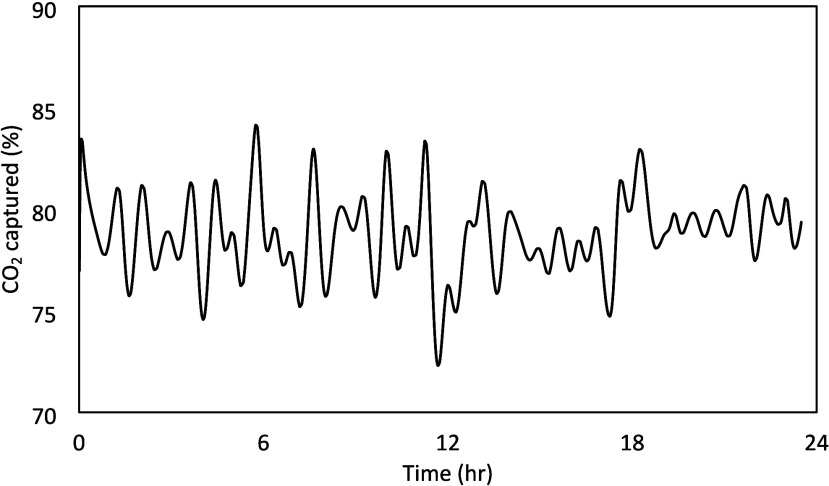
CO_2_ capture prediction from the Schroeder-phase input signals.

The power spectrum of the designed Schroeder-phase input signals is presented in Figure 11. Figure 12 presents the power spectrum for CO_2_ capture, which exhibits high and reasonably flat power over the entire frequency range of interest. It can be observed that the designed Schroeder-phased inputs have better properties in comparison to the PRBS signals, as the mean and minimum power/frequency for the CO_2_ capture corresponding to the Schroeder-phased inputs are about 51.1 and 35 dB/Hz, respectively, while those for the PRBS inputs are 40.5 and 20.7 dB/Hz, respectively. Standard deviations of power/frequency for the CO_2_ capture are similar for both types of signals (9.3 dB/Hz for PRBS and 9.7 dB/Hz for Schroeder-phased inputs). In addition, the Schroeder-phased input can be implemented in shorter time span than the PRBS signal. For the NCCC system, the PRBS signal took about 32 h to execute while the Schroder-phased inputs took less than 24 h to execute the entire sequence. It should be noted that if the PRBS signals are desired to be implemented within 24 h as well, then one option is to reduce the number of shift registers, but then the signal would not be persistently exciting for all orders.

**Figure 11:**
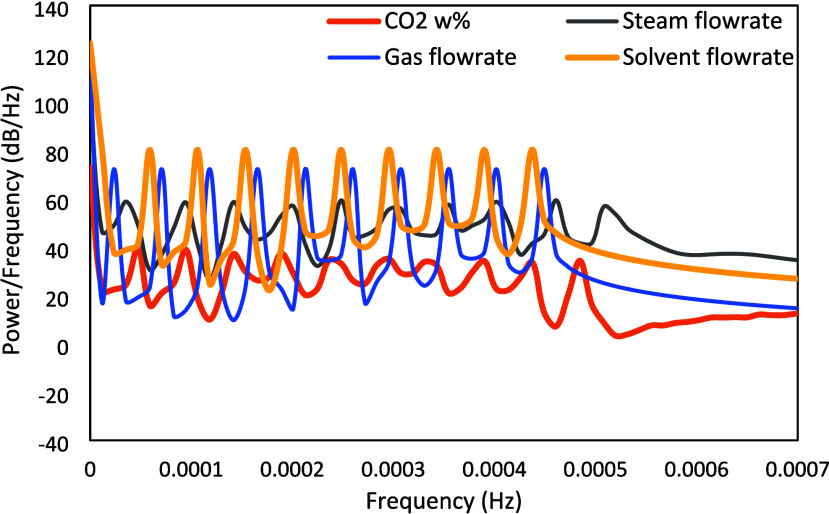
Power spectrum of the designed Schroeder-phased signals.

**Figure 12:**
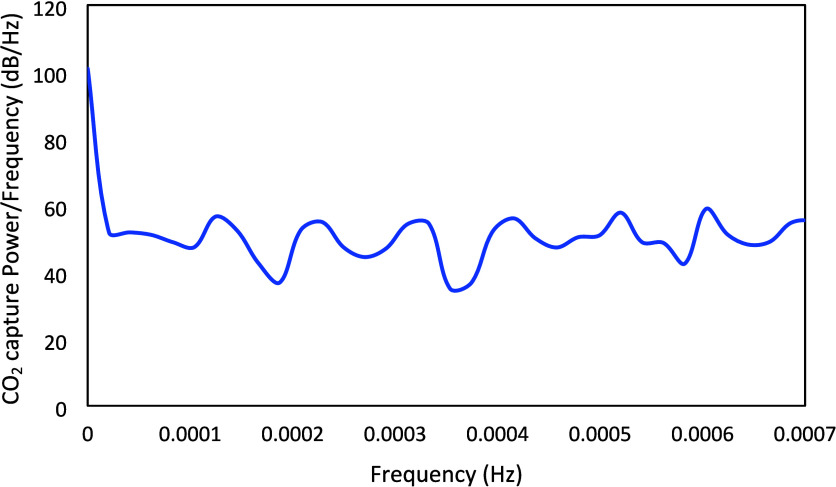
Power spectrum of the simulated CO_2_ capture corresponding to the designed Schroeder-phased signals.

## 4 Implementation Error

The signals designed and presented in the previous sections were implemented during early June in NCCC over the course of approximately 3 days (about 2 days for the PRBS DoDE followed by 1 day for running the Schroeder-phased DoDE). Environmental temperature variation during those days at the location of the pilot plant was less than 5 °C considering the average day- and nighttime temperatures. Also as shown in Figure 1, there are temperature controllers at key locations on the solvent streams. Thus, the impacts of variation in the environmental temperature on the test runs are considered to be negligible. The PRBS tests were started after the plant was brought close to the desired steady state. After the PRBS tests were completed, the plant was brought back close to the desired steady state before the Schroeder-phased DoDE. Figure 13 presents the designed PRBS and the actual implementation and measured values for the main input variables discussed in Section 2. It is observed that there is low implementation error in the solvent flow rate and steam flow rate, but there is high implementation error in the gas flow rate, which can be mainly attributed to the blower control system that plays a key role in varying the inlet gas flow rate to the absorber. The main discrepancy is found in the implementation of the input signal for wt % CO_2_. The actual value did never reach below about 12.5 wt % CO_2_ while the desired value was as low as 10 wt % CO_2._ This could be attributed to the limitations in the dilution air flow rate and considerable variation in the CO_2_ composition from the host power plant. Figure 14 compares the power spectrum of the actual vs design signals for the CO_2_ weight percentage in the flue gas. It is observed that the implemented signal still has reasonably high power in the entire range. The standard deviation and minimum power/frequency for the CO_2_ wt % in the flue gas corresponding to the designed PRBS signals were 7.3 and 23.3 dB/Hz, respectively, while those for the actual signal were 8.6 and 17 dB/Hz, respectively.

**Figure 13:**
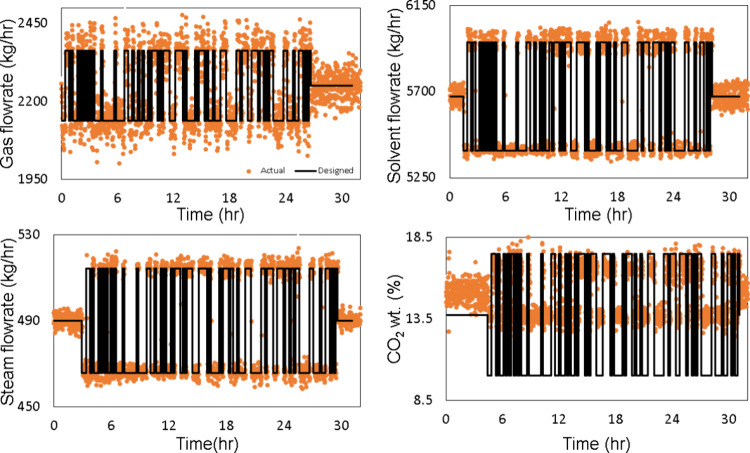
Implementation error for the PRBS.

**Figure 14:**
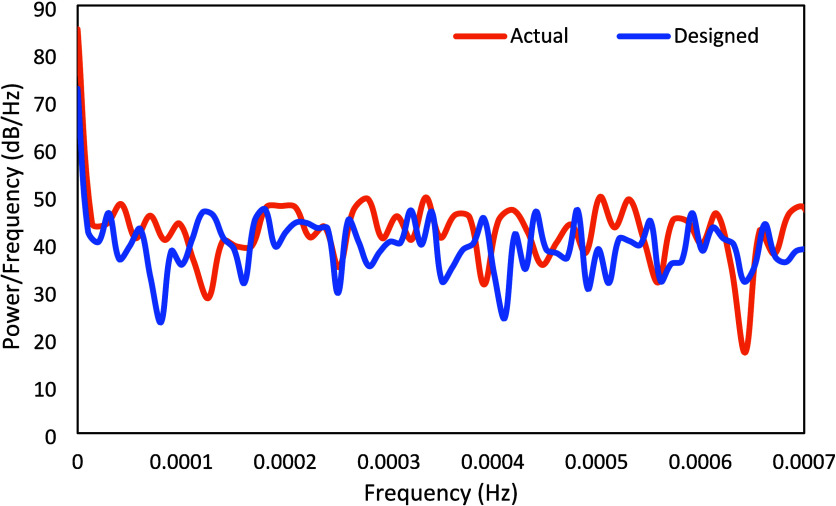
PRBS power spectrum density for the CO_2_ wt %.

Figure 15 shows that the implementation error in the Schroeder-phased input signals is far less than the PRBS signals with the exception of the CO_2_ wt % in the flue gas, even though all signals are noisy. Figure 16 compares the power spectrum of the actual vs design signals for the CO_2_ weight percentage in the flue gas. It is observed that the implemented signal still has a reasonably high power over the entire range. The standard deviation and minimum power/frequency for the CO_2_ wt % in the flue gas corresponding to the designed Schroeder-phased input signals were 12.3 and 3.3 dB/Hz, respectively, while those for the actual signal were 11.2 and 5.4 dB/Hz, respectively.

**Figure 15:**
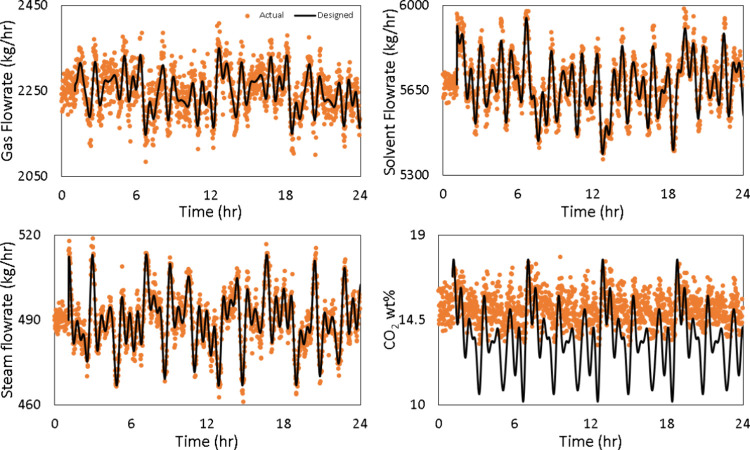
Implementation error for the Schroeder-phased input.

**Figure 16:**
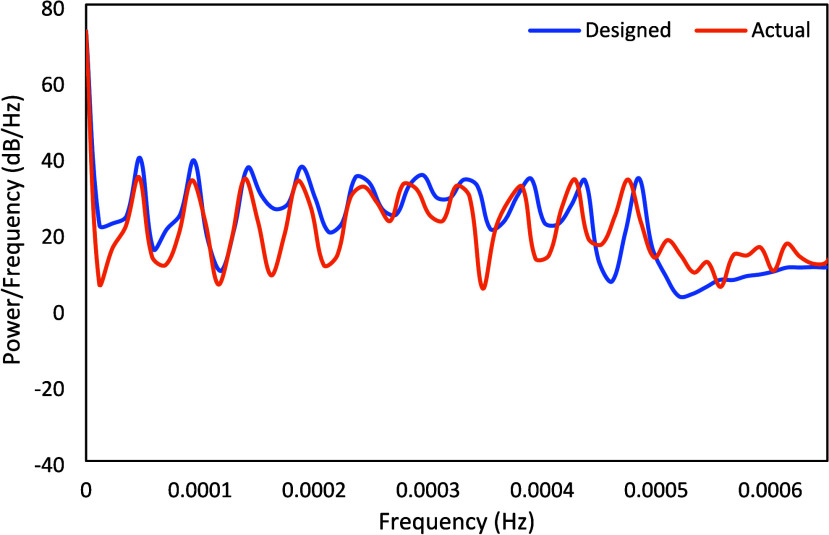
Schroeder-phased input power spectrum density for the CO_2_ wt %.

It may be feasible to reduce the nonuniformity of the power spectrum profile in Figures 14 and 16 by redesigning the input signals with due consideration of implementation error as well as plant-friendliness. However, such an approach is expected to require multiple iterations since the implementation error can again change for any newly developed input signal design. Since for both PRBS and Schroeder-phased input signals there is still high power in the entire range, such an attempt was not deemed necessary.

## 5 Dynamic Data Reconciliation (DDR) and Parameter Estimation

The raw data from the NCCC pilot-plant are preprocessed by applying a Butterworth filter (a band-pass filter) followed by a moving-average filter to remove noise from the data. The filtered data are then utilized for dynamic data reconciliation (DDR) and parameter estimation. The DDR methodology is similar to the approach in our previous work,^23^ but in this work a parameter estimation problem is also solved since the data contain required properties for parameter estimation. It is desired that parameters corresponding to the mass transfer coefficients, interfacial area, and holdup be estimated since they have most impact on the process performance including the transient response and are packing specific.^28^ While VLE, and other thermo-physical properties also affect the performance, they are not expected to be packing specific and those models were developed and calibrated by using available experimental data in the public domain as discussed earlier.^26,27^ However, as the dynamic model is developed in APD and therefore the optimization problem for parameter estimation must be solved in the APD platform by using the optimizers available in APD, only a sequential approach could be implemented. In the sequential approach, the optimizer does not directly consider the differential algebraic equation (DAE) system corresponding to the process model as constraints; rather, the DAE system is solved by the integrator. This sequential dynamic optimization problem led to convergence issues in APD. One issue with the sequential approach with such problems is that the nonlinear programming problem (NLP) solver cannot take advantage of the sparsity or structure of the underlying dynamic model, neither that of the KKT system.^38^ There are additional aspects that make this particular problem difficult to converge. First, the test runs took place over a large number of hours (about 24 h for the Schroeder-phased input signal and about 33 h for the PRBS signals). This leads to a significant computational cost for the DAE solver. It should be noted that the process model has more than 33,000 DAEs and thus the underlying dynamic optimization problem leads to more than 50 MM constraint equations even when the test data corresponding to the Schroeder-phased input signal is considered with 1 min uniform time step. In fact, both PRBS and Schroeder-phased signals cause steep changes in inputs and resulting output variables; thus, successful convergence of the highly nonlinear DAEs corresponding to the tower model requires very small time steps even less than 1 s at certain time instances, thus increasing the problem size manifold. Second, since a data reconciliation problem is also solved, it leads to a large number of decision variables. Third, as discussed earlier, the plant is not necessarily in the steady state when the signals are initiated. Therefore, the initial value of some of the key state variables are also estimated, leading to the large dimensionality of the problem. Fourth, there are some missing measurements such as the water makeup flow rate to the intermediate storage tank. These need to be estimated, as well. Fifth, when the parameters for the mass transfer coefficients, interfacial area, and holdup models are included in the estimation problem, it leads to a large dimensional optimization problem. Even with several days of simulation, the parameter estimation problem for not even a single model could be successfully solved. It should be noted that in comparison to the dynamic DDR problem solved earlier by the authors,^23^ here the dimensionality and computational expense of the problem became significantly larger due to inclusion of the initial values of some of the state variables and because of the long time span of the test run data. Since dynamic data reconciliation, initial state estimation, and missing measurement estimation must be considered, it was decided that the parameter estimation will be performed only for the holdup model since that has a strong impact on the transient response but can be difficult to accurately estimate by using the steady-state data. Furthermore, due to high implementation error and significant noise in the input data and due to the high noise in the output data corresponding to the PRBS design and due to high computational time required by the DAE solver for the PRBS design (the multistep Gear’s method in APD had to cut steps significantly due to steepness allowing only very short integration steps for convergence), it was desired that the parameter would be estimated by using the data corresponding to the Schroeder-phased input signal design. These estimated parameters are then tested on the data corresponding to the PRBS design.

### 5.1 DDR and Parameter Estimation Using the Data Generated by Schroeder-Phased Input Signals

The DDR with parameter estimation was implemented as an optimization problem in APD, as given by eq 27:

$$\begin{array}{l} \min{\left(y_{\exp}-y\right)}^{\prime }\sum^{-1}\left(y_{\exp}-y\right)\\ \mathrm{s.t.}\\ H\left(\eta ,y,u,\theta \right)=f\left(\eta ,y,u,\theta \right)\\ g\left(\eta ,y,u,\theta \right)\leq 0 \end{array}$$ 27

The following input variables are considered as decision variables: flue gas flow rate, lean solvent flow rate, steam flow rate, and CO_2_ concentration in the flue gas. These variables were also included while calculating the objective function. In addition, following variables were reconciled:

- Lean CO_2_ loading
- Gas flow rate from absorber
- CO_2_ concentration in the absorber outlet flue gas
- Lean solvent temperature to the absorber
- Lean solvent temperature from regenerator

The estimated parameters for the holdup model are presented in Table 1. These parameters correspond to the final holdup correlation used in our previous work.^28^ The correlation is given by eq 28:

$$h_{L}=H_{L1}{\left[\frac{1}{S^{2}g^{2/3}}{\left(\frac{\mu_{L}}{\rho_{L}}\right)}^{1/3}\frac{Au_{L}}{L_{P}}\right]}^{H_{L2}}$$ 28

**Table 1:** Estimated Holdup Parameters

parameter	original value^28^	Estimated value through dynamic DDR and parameter estimation
*H*_L1_	11.45	11.5
*H*_L2_	0.6471	0.39

It should be noted that the holdup correlation given by eq 28 was developed by Tsai,^39^ but its parameters *H*_L1_ and *H*_L2_ were optimally estimated in our previous work^28^ by using the available experimental data.^39^ The estimated value for the linear parameter *H*_L1_ did not change much compared to the original value estimated in our earlier work,^28^ while *H*_L2_ got reduced from approximately 0.65 to 0.39. It should be noted that these parameters for the holdup model were estimated in our earlier work^28^ by using the holdup data measured from an air–water system, i.e., a nonreactive system.^39^ Thus, accuracy of the original parameters is questionable for a reactive CO_2_ capture system. To the best of the authors’ knowledge, there are currently no measured data available in the open literature for reactive CO_2_ capture systems. The difficulties in measuring the holdup for reactive systems is spatial variation in holdup that would require localized measurement of holdup along the packing rather than estimating holdup globally by simply measuring sump liquid volume change as was done for the experimental data used in our previous work.^39^ Furthermore, for reactive systems, changes in the liquid volume due to reaction and changes in the spatial variation in temperature due to absorption need to be accounted for while measuring the holdup. These difficulties motivate estimating the parameters for the holdup model by using the dynamic data, since the transient response would be affected by holdup as discussed earlier. It is also interesting to note that the column hydraulic parameters estimated in this work could be accomplished by using the dynamic data collected in about 24 h. In contrast, the data that were used for estimating the original parameters was collected by conducting hundreds of steady-state test runs on a nonreactive (water–air) system with variations in the water flow rate, air flow rate, air and water temperature, viscosity (by using an additive), and packing height.^39^ It is worth noting that the data obtained from the PRBS signal and Schroeder-phase input signal yield similar values of these parameters.

The discrete-time cumulative root means squared error (DCRMSE) for % CO_2_ capture is given by eq 29:

$$\mathrm{DCRMSE}=\sqrt{\frac{1}{N}\sum_{i=1}^{N}\varepsilon^{2}}$$ 29

Table 2 shows the DCRMSE for the original holdup model parameters^28^ versus the regressed holdup model parameters in this work by using the data obtained from the PRBS and Schroeder-phased input signals. Results show that the parameters obtained by using the dynamic data collected in this work for the actual reactive CO_2_ capture system are superior to the previous work^28^ even though they are not statistically too different. However, the key take-aways are that these parameters are estimated for the actual MEA-H_2_O–CO_2_ system and therefore a designer would have higher confidence in them as opposed to those obtained by using data from a nonreactive system and that the data needed for estimating these parameters could be obtained in considerably lesser time than the expected time to conduct hundreds of test runs on a nonreactive system.^39^ Furthermore, those test runs were especially conducted for measuring only the hydraulic data (i.e., pressure drop and holdup data) for a packed tower,^39^ while the dynamic data collected in this work could have been also utilized for development and parameter estimation of the mass transfer coefficient and interfacial area models. As noted above, these later tasks could not be undertaken due to software and other limitations discussed before.

**Table 2:** DCRMSE (% CO_2_ capture) for Original and Regressed Holdup Parameters

	original holdup parameters	regressed holdup parameters
pseudorandom binary signal	3.25	3.11
Schroeder-phased input signal	2.15	1.96

Figure 17 shows the measured versus reconciled Schroeder-phased inputs for the solvent flow rate, flue gas flow rate, CO_2_ concentration in the flue gas, and steam flow rates, respectively. Figure 18 presents the comparison between the model results and reconciled experimental data for the CO_2_ capture percentage.

**Figure 17:**
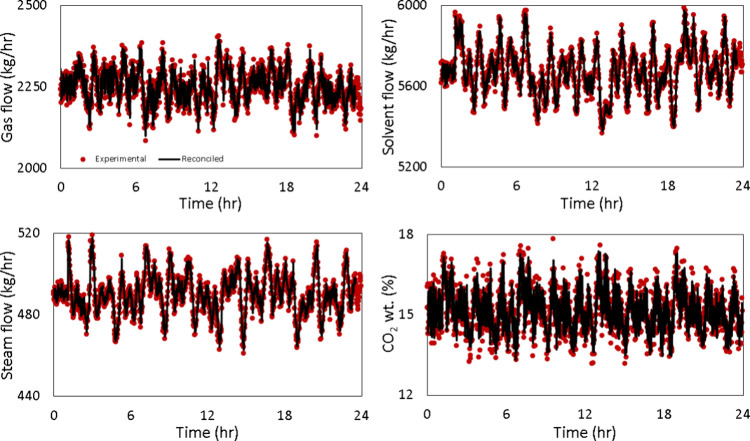
Reconciled vs measured Schroeder-phased input signal.

**Figure 18:**
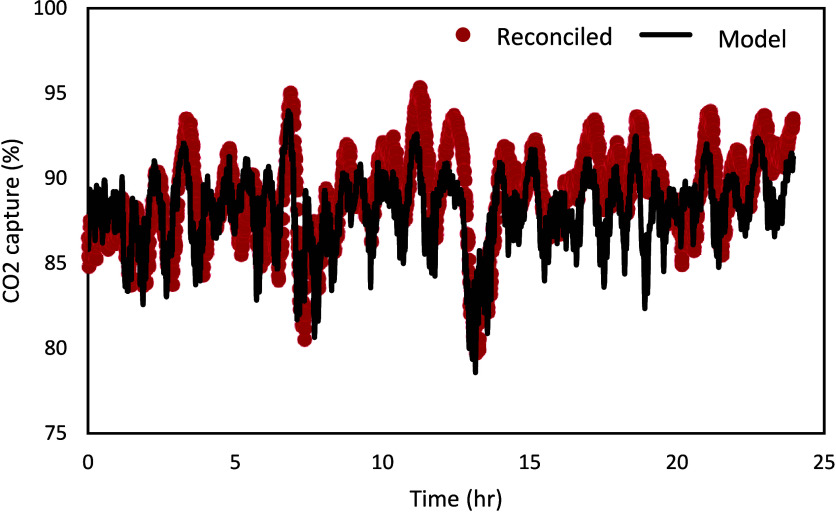
Model results vs reconciled experimental data for CO_2_ capture percentage for the Schroeder-phased input signal.

It is observed in Figure 18 that while the model could capture the general trend well, there are still opportunities for improvement. It can be noted that the solvent concentration, one of the key input variables, was not reconciled since a real-time measurement of solvent concentration was not available. In NCCC, solvent amine concentration was measured periodically (every 70 min) using an automated titration system. As noted in one of our earlier publications,^10^ the estimated expanded uncertainty (∼95% confidence, k = 2) of the online amine concentration is approximately 6%. The authors feel that reconciling the solvent concentration, if a real-time reliable measurement would have been available, and estimation of other parameters of other key models, particularly that of mass transfer coefficients and interfacial area, as noted earlier. could have improved the estimation accuracy. It is worth noting that while it is desired to maintain the solvent concentration nearly constant, it is challenging to maintain it during dynamic test runs due to significant variations in the operating conditions. In addition, for maintaining the concentration, real time measurement of the solvent concentration is desired, which is typically not available in pilot plants given the limitations of current measurement technology.

### 5.2 DDR Using the Data Generated by PRBS

The dynamic data generated by PRBS are also reconciled. Figure 19 shows the experimental versus reconciled PRBS for the solvent flow rate, flue gas flow rate, CO_2_ concentration in the flue gas, and steam flow rates, respectively.

**Figure 19:**
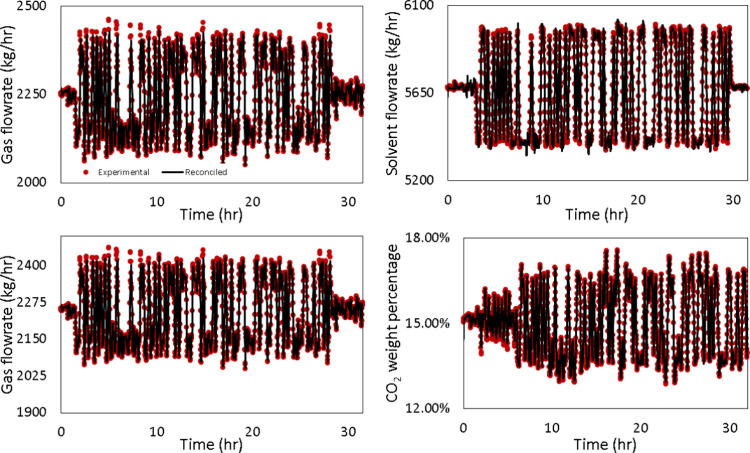
Reconciled vs measured PRBS signal.

Figure 20 compares the model results with the reconciled experimental data for CO_2_ capture. Similar to the results for the Schroeder-phase input signals, although the model could capture the trend generally well, there are certainly opportunities for improvement. Similar to the case before, the authors feel that reconciling the solvent concentration using a real-time reliable measurement and estimation of other model parameters could have improved the estimation accuracy. Considerable implementation errors also contribute to the error.

**Figure 20:**
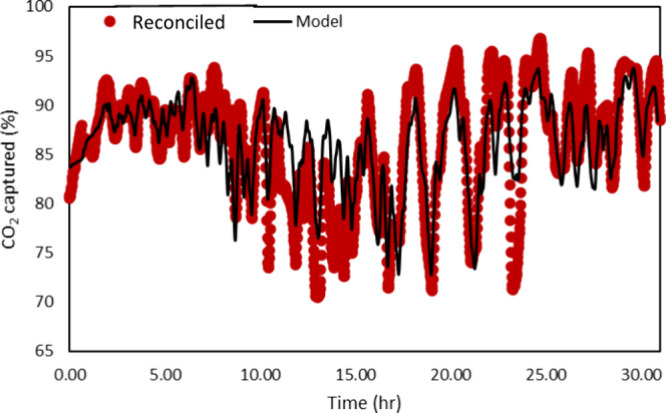
Model results vs reconciled experimental data for the CO_2_ capture percentage for the PRBS input signal.

### 5.3 Case Studies

Figure 21 shows the transient response of CO_2_ capture as a result of +5% step changes in the lean solvent flow rate and the flue gas flow rate. Here the regressed holdup parameters are used. “Case a” presents a −5% solvent flow rate step change, and “case b” shows a +5% solvent flow rate step change. The same percent step changes are implemented for the flue gas flow rate in cases “c” and “d”, respectively. These results are generated by considering only the absorber model, and therefore, the inlet solvent concentration and loading remain unchanged (i.e., dynamics of the remaining equipment including stripper dynamics and dynamics of the steam flow controller are not taken into consideration).

**Figure 21:**
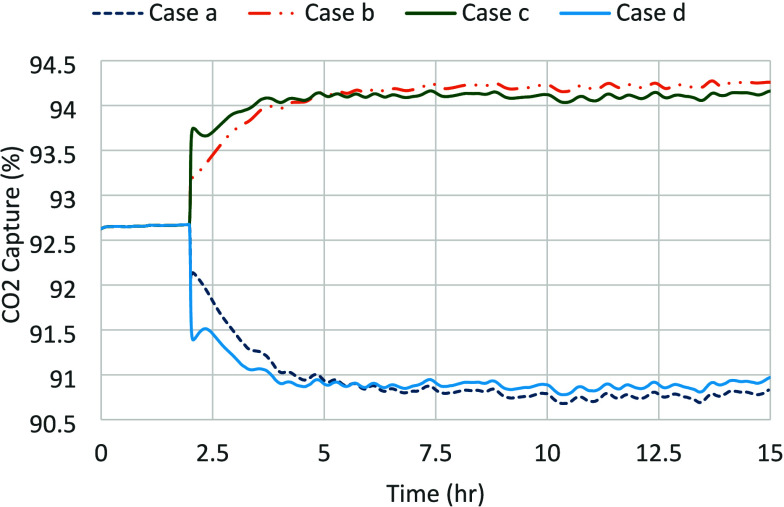
– Transients of the CO_2_ capture response for Case a (5% decrease in the lean solvent flow rate), Case b (5% increase in the lean solvent flow rate), Case c (5% decrease in the flue gas flow rate), Case d (5% increase in the flue gas flow rate).

First of all, the CO_2_ capture response for the change in the solvent flow rate is closer to that of the flue gas flow rate in comparison to what has been observed earlier with the original holdup model parameters.^28^ In addition, the gains are found to be 0.36 and 0.33% CO_2_ capture/% solvent flow rate, respectively when the solvent flow rate is increased and decreased, respectively, as expected, as opposed to 0.58 and 0.46% CO_2_ capture/% solvent flow rate, respectively, using the original holdup model parameters.^28^ Similar observations are made for the response when the flue gas flow rate is changed. There is practically no difference in the time constant between the earlier and the current responses.

## 6 Conclusions

This paper describes the design and implementation of PRBS and Schroeder-phase input signals for a CO_2_ capture pilot plant. Signals are designed for a multivariable system ensuring output plant friendliness. The Schroeder-phased input signal took about 9 h less time than the PRBS design. Both input signal designs result in the power of the signals reasonably uniformly distributed over the entire frequency range of interest. However, the designed Schroeder-phase inputs yield higher mean and minimum power/frequency for the CO_2_ capture in comparison to those of the PRBS signal. Higher implementation error is observed for the PRBS. Considerable implementation error is observed in the signal for the CO_2_ concentration in the flue gas for both types of input signals. In spite of the implementation error, the power spectrum of the actual signal is still reasonably close to that of the designed signals albeit low power at some frequency ranges.

A sequential strategy is implemented for solving the DDR and parameter estimation problem. A number of key input and output variables are reconciled so that plant mass and energy balances could be satisfied. A few key unmeasured input variables are also estimated. Due to computational intractability, the parameter estimation problem considered the estimation of only the holdup model parameters. While the model predicted the transient response well, especially for the Schroeder-input signal, there are certainly opportunities for reducing the discrepancy between the data and the model. The authors felt that reconciling the solvent concentration using a real-time reliable measurement and estimation of parameters of models other than the holdup model could have improved the estimation accuracy. The study shows that the DDR and parameter estimation approach can yield parameter estimates for the actual system by using data collected in considerably less time than traditional steady-state test runs. In the particular case of the holdup model parameters, the estimated parameters are not only superior to those obtained from using data collected from hundreds of steady-state test runs in a nonreactive (air–water) system, but they could be obtained using the data collected for about 24 h for the actual MEA-H_2_O–CO_2_ system. The authors believe that DoDE can be instrumental in reducing the experimental time considerably compared to steady-state test runs. Furthermore, DoDE can yield information such as those related to holdup of mass/energy/momentum that is difficult to obtain from the steady-state test runs.

It is observed that when the updated holdup parameters are used, then the gain in the transient response of CO_2_ capture as a result of change in the solvent flow rate is about 60–70% to that when the original holdup model parameters are used. Since the solvent flow rate is a key manipulated variable and flue gas flow rate is a key disturbance variable, an accurate estimate of these gains can be helpful in the design of efficient model-based control systems.

While further improvement in the results is anticipated if computational capability can be developed in the future for simultaneous estimation of other model parameters and dynamic data reconciliation, this paper shows the promise of DoDE.
